# Functional richness shows spatial scale dependency in *Pheidole* ant assemblages from Neotropical savannas

**DOI:** 10.1002/ece3.5672

**Published:** 2019-09-27

**Authors:** Karen Neves, Mario R. Moura, Jonas Maravalhas, Renata Pacheco, Marcio R. Pie, Ted R. Schultz, Heraldo L. Vasconcelos

**Affiliations:** ^1^ Instituto de Biologia Universidade Federal Uberlândia Uberlândia MG Brazil; ^2^ Department of Ecology and Evolutionary Biology Yale University New Haven CT USA; ^3^ Departamento de Zoologia Universidade Federal do Paraná Curitiba PR Brazil; ^4^ Department of Entomology National Museum of Natural History Smithsonian Institution Washington DC USA

**Keywords:** ant richness, Brazilian Cerrado, dominant ant, ecological packing, functional richness, spatial scale, species assemblage

## Abstract

There is a growing recognition that spatial scale is important for understanding ecological processes shaping community membership, but empirical evidence on this topic is still scarce. Ecological processes such as environmental filtering can decrease functional differences among species and promote functional clustering of species assemblages, whereas interspecific competition can do the opposite. These different ecological processes are expected to take place at different spatial scales, with competition being more likely at finer scales and environmental filtering most likely at coarser scales. We used a comprehensive dataset on species assemblages of a dominant ant genus, *Pheidole*, in the Cerrado (savanna) biodiversity hotspot to ask how functional richness relates to species richness gradients and whether such relationships vary across spatial scales. Functional richness of *Pheidole* assemblages decreased with increasing species richness, but such relationship did not vary across different spatial scales. Species were more functionally dissimilar at finer spatial scales, and functional richness increased less than expected with increasing species richness. Our results indicate a tighter packing of the functional volume as richness increases and point out to a primary role for environmental filtering in shaping membership of *Pheidole* assemblages in Neotropical savannas.

**OPEN RESEARCH BADGES:**

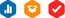
This article has been awarded Open Materials, Open Data, Preregistered Research Designs Badges. All materials and data are publicly accessible via the Open Science Framework at https://doi.org/10.5061/dryad.31201jg

## Graphical abstract

*Pheidole* ant assemblages from Neotropical savannas are more functionally similar at coarser than finer spatial scales. Our findings point out to a tighter packing of the functional volume of ant assemblages as richness increases, suggesting the primary role of environmental filtering in shaping membership of *Pheidole* assemblages in Neotropical savannas.

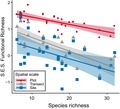

## 1 INTRODUCTION

Functional richness—the overall trait difference found in a given community—often relates monotonically with species richness (Petchey & Gaston, 2002). However, species richness variation may increase functional richness more or less than expected by chance depending on how ecological processes shape the community membership. For instance, if communities are saturated with species, then the limiting similarity theory predicts that higher species richness translates into greater filling of the “niche space” (MacArthur & Levins, 1967). That is, competition would lead co‐occurring species to explore different resources and likely exhibit niche differences that ultimately increase functional richness more than expected given the local species richness. Environmental filtering should act in the opposite direction and add functionally similar species to communities, leading functional richness to increase less than expected for a given species richness (Swenson & Weiser, 2014). Alternatively, if community composition is not constrained by deterministic ecological processes, then the functional richness might show random deviations along richness gradients, likely reflecting the role of stochastic processes in community assembly (Cavender‐Bares, Kozak, Fine, & Kembel, 2009).

The relative importance of these deterministic ecological processes, such as interspecific competition and environmental filtering, is hypothesized to vary with the spatial scale (Ricklefs & Jenkins, 2011). Consequently, the relationship between species richness and functional richness may also differ across scales. Negative interactions such as competition take place at very small spatial scales where environment is more homogeneous and encounters between organisms are more likely (Araújo & Rozenfeld, 2014). Conversely, abiotic filters would leave stronger signatures on communities at coarse spatial scales, since increasing area is usually accompanied by increases in environmental heterogeneity that allows sorting of ecologically similar species in contrasting environments (Weiher & Keddy, 1995). Under the functional perspective, studies have shown plant communities composed of more functionally dissimilar species at finer spatial scales, with the opposite taking place in communities assembled at coarser scales (Cavender‐Bares, Keen, & Miles, 2006). However, the sparse evidence available on the scaling of functional richness or phylogenetic richness—its analogous form if we assume niche conservatism—has been inconsistent for animal communities (Gómez, Bravo, Brumfield, Tello, & Cadena, 2010). Overall, most cross‐scale studies have focused on plants and vertebrates, with the spatial scaling of functional richness of invertebrates remaining virtually unexplored.

We investigate the spatial scaling and richness dependency of functional richness of *Pheidole* ant assemblages in the Cerrado (savanna) biodiversity hotspot in South America. *Pheidole* is one of the richest ant genera in the world and known to be particularly dominant in tropical habitats (Economo et al., 2015; Wilson, 2003). Since Cerrado ant richness varies strongly with latitude and climate (Vasconcelos et al., 2018), strong variation is expected in the number of *Pheidole* species coexisting in local savanna ant assemblages. Interspecific competition is also expected to be higher among congeneric species due to their potential similar ecological roles. *Pheidole* ants are thus an ideal group to elucidate processes underlying community assembly across spatial scales and along richness gradients. If interspecific competition plays a strong role in shaping community membership, we expect functional richness to increase more than expected with increasing species richness; with such an effect being stronger at finer than coarse spatial scales. Conversely, if environmental filtering has higher relative importance in assembling ant species, then functional richness will increase less than expected with increasing richness; particularly at coarser spatial scales.

## 2 MATERIALS AND METHODS

### 2.1 Species assemblage data

We sampled 29 Brazilian sites distributed in the Cerrado, the largest savanna region in South America. Our sampling sites were restricted to *cerrado* sensu *stricto* areas, a dominant Cerrado physiognomy characterized mainly by a mixture of shrubs and sparse woody vegetation (Oliveira‐Filho & Ratter, 2002). At each site, we established three 380‐m long linear transects, each of which was at least 1 km from the nearest neighboring transect (median distance among transects in each site was 3 km). Along each transect, we installed 10 plots distributed at 40‐m intervals. Each plot consisted of four non‐baited pitfall traps arranged in a grid of approximately 2.5 × 2.5 m that remained in operation for 48 hr. Overall, the hierarchical spatial scale of this study is summarized in 870 plots of 6.25 m^2^ distributed along 87 transects (each one covering an area of ca. 1,000 m^2^) located in 29 sites (each site covering an area of ca. 9 km^2^). All sampled ants were separated into morphospecies (hereafter, “species”) by the same person (K. Neves) and then identified using the available bibliography (Wilson, 2003) and/or through comparison with ant species deposited in the entomological collections of the Universidade Federal de Uberlândia (UFU) and Universidade Federal do Paraná (UFPR), both in Brazil. Voucher specimens were deposited in these same collections.

### 2.2 Functional traits

The biology of most ant species is poorly known. Consequently, most studies that focused on ant functional traits have relied on morphological traits with known or presumed ecological functions in ants (Liu, Guénard, Blanchard, Peng, & Economo, 2016; Santoandré, Filloy, Zurita, & Bellocq, 2019). Although we sampled ant species of all genera, we measured functional traits only for *Pheidole* species due to their high dominance among species occurrence in our samples. We quantified five morphological traits commonly investigated in ants that often relate to specialization in species foraging and habitat complexity (Guilherme et al., 2019; Silva & Brandão, 2010). The hypothesized functions of such morphometric traits are linked to metabolism, resource acquisition, trophic position, and/or habitat use of the species (for further details on these traits and their presumed functions, see Parr et al., 2017; Santoandré et al., 2019; Weiser & Kaspari, 2006). We measured the following: (a) Weber's length (WL)—the maximum length from the anterior edge of the pronotum to the posterior edge of the propodeum. WL is a measure of total body size and correlates with metabolic and dietary characteristics. (b) Eye length (EL)—the maximum diameter of the eye. EL is indicative of habitat use, food searching behavior, and activity times. (c) Femur length (FL)—the maximum length from the base (including trochanter) to the tibia insertion. FL is correlated with foraging speed and indicative of thermoregulatory strategy. (d) Mandible length (ML)—the maximum length from the basal margin to the apical tooth of the mandible. ML relates to type of diet as predatory species have longer mandibles than omnivorous species. (e) Scape length (SL)—the maximum length from the base to the apex of the scape. SL is indicative of sensory abilities in ants, including especially the ability to follow chemical cues or to detect resources. Whenever possible, we used at least five specimens (range = 5–20) to take the mean trait measures for each *Pheidole* species. However, for 46 rare species, trait measures were based on the one or two specimens available. In total, 356 specimens from 98 species or morphospecies were measured. All trait measures were carried out by the same person (K. Neves), using an ocular micrometer on a stereo microscope. Trait data were taken only from minor workers to maximize coverage over species in our samples. EL, FL, ML, and SL were measured as the ratio between the respective trait and WL. A recent study showed that the traits we measured present low levels (1%–4%) of intraspecific variation (Gaudard, Robertson, & Bishop, 2019); therefore, we assumed functional traits to be species‐specific and the inter‐site variability to be negligible.

### 2.3 Quantification of functional richness

Measures of functional richness can be either extracted from the multidimensional functional space representing the species in a sample (Villéger, Mason, & Mouillot, 2008) or based on branch lengths derived from functional dendrograms (Petchey & Gaston, 2007). Although the building of a dendrogram from a distance matrix leads to the loss of some information, it allows the computation of functional metrics for species‐poor assemblages, in contrast to the Villéger et al. (2008) approach, which requires the number of species in a sample to be higher than the number of functional traits (five in this study). Considering that 800 of the 870 plots and 14 of the 87 transects hold five or less *Pheidole* species, we used only the dendrogram‐based approach to compute functional richness metrics. To do so, we initially carried out a principal component analysis (PCA) on the standardized trait data (mean = 0, *SD* = 1) to obtain orthogonal (uncorrelated) trait axes. We then used these PCA axes to compute the pairwise Euclidean distance between each *Pheidole* species. The Euclidean distance matrix was passed through a UPGMA clustering analysis to produce a functional dendrogram, following recommendations by Podani and Schmera (2007).

We used the sum of the branch lengths across species in each assemblage to quantify the functional richness (FR, Petchey & Gaston, 2002). To verify whether functional richness differed from the values expected given the species richness of a sample, we built a null distribution of FR values based on 1,000 randomizations of species across the tips of the functional dendrogram, while holding the internal structure of the species‐by‐site matrix constant (e.g., species‐occurrence frequency, species richness, and co‐occurrence patterns; Swenson, 2014). We then computed the standardized effect size of functional richness (SES.FR). SES.FR values higher than expected indicate that functional richness increases more than expected given the species richness, with an opposite interpretation for lower than expected SES.FR values.

All measures of functional richness (FR and SES.FR) were computed using plots, transects, and sites as the basic sampling unit. Only sampling units with at least two species were used in the analyses, resulting in the exclusion of 123 out of the 870 sampling plots from computations of functional richness at the plot level. All sampling units were retained at the transect and site levels. Computations were performed in R 3.3.3 using the *picante* (Kembel et al., 2014) package.

### 2.4 Data analysis

The hierarchical spatial design of our study inevitably results in a highly unbalanced number of sampling units at each level of spatial scale (*n* = 870 plots, *n* = 30 transects, *n* = 29 sites). To make our response variable (standardized effect size of functional richness, SES.FR) more comparable across the different spatial scales, we averaged the measures of functional richness of plots and transects within a same site to get the average functional richness at the plot scale (*n* = 29) and transect scale (*n* = 29), respectively. These averaged measures were used to compare how the SES.FR vary across the three spatial scales (*n* = 29 for each spatial scale). We used Kruskal–Wallis tests to verify whether the median of a given response variable differed across the spatial scale levels (plot, transect, site). As mentioned earlier, ecological processes such as environmental filtering and competition can change the relationship between functional richness and species richness. If these ecological processes left signatures on *Pheidole* assemblages, we expect to find positive (supporting competition) or negative (supporting environmental filtering) associations between *Pheidole* richness and SES.FR. To test this hypothesis, we regressed each SES.FR measure against the *Pheidole* richness observed at each site. To control for spatial correlation in the response variables and *Pheidole* richness, we used a modified *t* test (Dutilleul, 1993) to correct the degrees of freedom of correlation coefficients. Computations were performed in R 3.3.3 using the packages *SpatialPack* (Osorio, Vallejos, & Bevilacqua, 2018) and *pgirmess* (Giraudoux, 2018).

## 3 RESULTS

We recorded 2,650 species‐occurrence records for 98 species of *Pheidole* ants (Table S1). The median *Pheidole* richness per site was 16 species (range = 6–32). At the spatial scale of transects, the *Pheidole* richness ranged from 3 to 22 (median = 9), and at the plot scale, it varied from 1 to 11 species (median = 3). The overall ant richness (measured at the spatial scale of sites) was strongly correlated with *Pheidole* richness (*r* = .844, spatially corrected *df* = 8.833, *p* = .001).

**Figure 1:**
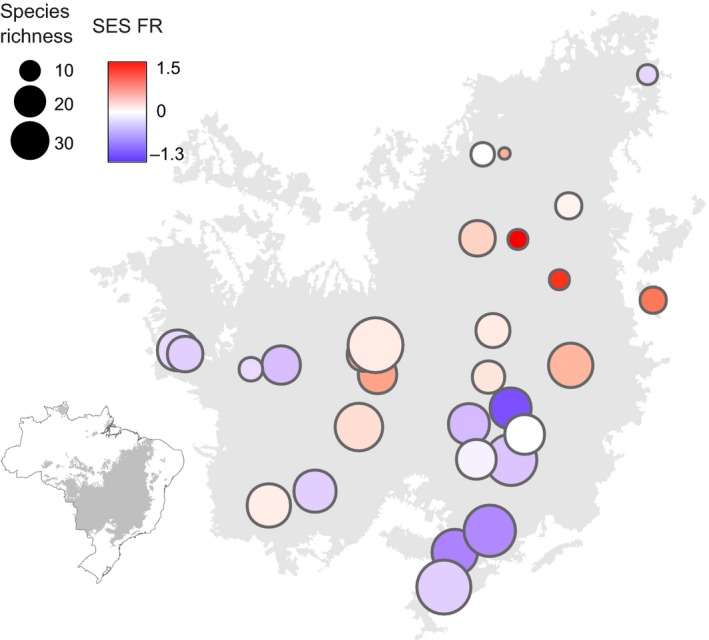
Geographic pattern of functional richness for *Pheidole* assemblages in the Cerrado, Brazil. The color gradient shows values of the standardized effect size of the functional richness (SES.FR) whereas circle symbol size is proportional to the *Pheidole* species richness of each site

**Figure 2:**
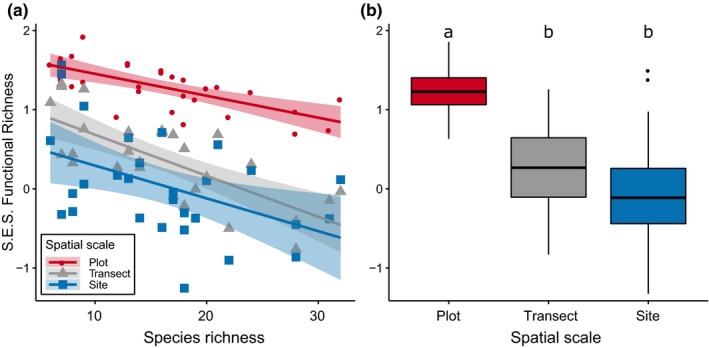
Spatial scaling of functional richness (SES.FR) of *Pheidole* assemblages along richness gradients in the Cerrado. Each box denotes the median (horizontal line) and the 25th and 75th percentiles. Vertical lines denote the 95% confidence intervals, and black dots are outliers. Small capital letters denote the results of the Kruskal–Wallis tests for the difference in medians across different spatial scales (*p* = .05, using Bonferroni correction)

*Pheidole* species richness decreased with latitude whereas the standardized effect size of functional richness (SES.FR) tended to increase (Figure 1). The relationship between the SES.FR and species richness was negative in all three spatial scales (Figure 2a). After correcting *p*‐values for the presence of spatial autocorrelation, such relationship remained significant at the spatial scale of transects (*r* = −0.718, *p* = .011) and sites (*r* = −0.687, *p* = .008), but not at the plot scale (*r* = −0.469, *p* = .076). The slopes of the relationship between SES.FR and *Pheidole* richness did not differ across spatial scales (likelihood‐ratio test between models with and without the interaction term, *χ* ^2^ = 0.453, *df* = 2, *p* = .283). However, the median SES.FR differed across spatial scales (Kruskal–Wallis *χ* ^2^ = 46.3, *df* = 2, *p* < .001), with species assemblages showing more functionally dissimilar ants at the spatial scale of plots rather than transects or sites (Figure 2b).

## 4 DISCUSSION

We sought to answer how the functional richness of *Pheidole* assemblages varies across spatial scales and along richness gradients. The accumulated evidence from theoretical and observational studies (mostly on plants) indicates that species assemblages are functionally overdispersed at small scales and clustered at coarser spatial scales (Cavender‐Bares et al., 2006; Swenson, Enquist, Pither, Thompson, & Zimmerman, 2006). Our findings agree with those of previous studies. The evolutionary lability of *Pheidole* traits (Economo et al., 2015), in concert with the lower than expected functional richness in species‐rich *Pheidole* assemblages, points to the greater role of niche convergence in shaping ecological adaptations of *Pheidole* ants to the abiotic environment of Neotropical savannas.

The cross‐scale variation in functional richness (SES.FR) of *Pheidole* assemblages is in line with local studies on niche partitioning. Analysis of species co‐occurrence patterns shows that ant species segregate in space more than expected at finer spatial scales but distribute themselves randomly at coarser scales (Albrecht & Gotelli, 2001), or are randomly distributed at finer scales but aggregated at coarser scales (Fowler, Lessard, & Sanders, 2014; Sanders et al., 2007). Such spatial niche partitioning is often associated with competitive structuring of ant assemblages, and it is more common among the dominant ant species (Parr & Gibb, 2010). In this study, *Pheidole* is the most dominant ant genus (greatest number of species occurrences) in 88% of plots, 81% of transects, and 86% of sites (H. L. Vasconcelos, personal observation), and it was always among the five most common genera in any given plot, transect, or site. Thus, the higher values of the SES.FR at the plot scale (6.25 m^2^) likely reflect the greater niche partitioning of *Pheidole* ants at very small spatial scales. Although competition may be important at small spatial scales, its role in structuring ant assemblages can decline as habitat complexity increases (Cerdá, Arnan, & Retana, 2013; Sarty, Abbott, & Lester, 2006). Above a given spatial scale, biotic interactions might lose importance in shaping the species pools, with broad‐scale environmental filters (e.g., temperature, precipitation) and/or regional factors driving the community assembly processes at coarser spatial scales (Lessard et al., 2012). The functional traits we investigated likely affect colonization of *Pheidole* ants up to 1,000 m^2^ of spatial scale. Above this scale, functional (morphometric) richness practically does not change, indicating that other functional traits become important at coarser scales (e.g., thermal tolerance).

Our findings indicate a tighter packing of the functional volume as richness increases. This ecological packing can be due to at least three factors. First, interspecific competition is weak and *Pheidole* species show greater niche overlap due to environmental filtering. There is some evidence that trophic niche breadth in ants is roughly constant, with trophic niche overlap being greater than expected (Fowler et al., 2014) and positively related to ant richness (Bernstein, 1979). Moreover, ant assemblages from highly productive regions can show greater spatial niche overlap (Segev, Kigel, Lubin, & Tielbörger, 2015) and fewer behaviorally dominant species (Arnan, Cerdá, & Retana, 2014). The higher productivity found in the southeastern Cerrado, where *Pheidole* assemblages are more functionally packed, may reflect greater food availability there, allowing the coexistence of ant species with similar resource needs. Indeed, studies at the Cerrado savannas indicate a positive association between ant richness and both richness and density of trees (Ribas, Schoereder, Pic, & Soares, 2003; Vasconcelos et al., 2019). Recent studies using the same ant dataset explored here (including all ant species and not only *Pheidole*) show that ant richness is correlated with both summer precipitation and net primary productivity but not with annual mean temperature (Vasconcelos et al., 2018), further reinforcing the potential role of productivity on Cerrado ant richness. Future research on patterns of dominance and co‐occurrence of ants along gradients of productivity may shed light on this question.

Second, competition is important, but it may interact with environment to allow finer partitioning of the “niche space” by ants in richer regions. Under more favorable conditions, ant species can forage at different periods and show greater temporal niche partitioning due to distinct temperature preferences (Albrecht & Gotelli, 2001; Cerdá, Retana, & Cros, 1998). Indeed, ant assemblages experiencing warmer and moister conditions show greater susceptibility to invasion by exotic ants than assemblages experiencing warm and dry conditions, suggesting that under particular abiotic conditions interactions among native ants become less important (Holway, Suarez, & Case, 2002; Menke & Holway, 2006). The Cerrado shows large differences in climatological regimes, with dry summers and rainy winters in the north and rainy summers and dry winters in the south. The concomitant warm and dry conditions in the northern Cerrado might restrict foraging times of tropical ants and increase temporal niche overlap (and thus competition). Such thermal constraints on ant species have been demonstrated even for temperate habitats, where thermal resilience of ant assemblages is reduced in warm and aseasonal regions (Arnan, Blüthgen, Molowny‐Horas, & Retana, 2015). Considering that tropical ant species are already close to their upper thermal tolerances (Diamond et al., 2012), it is likely that physiological limitations shape the outcome of the biotic interactions. Similar differences in climatological regimes have been used to explain the phylogenetic clustering of tropical vertebrate ectotherm assemblages facing rainy summers in contrast to the phylogenetic overdispersion of species assemblages experiencing dry summers (Moura, Costa, Argôlo, & Jetz, 2017). Although temperature and humidity are often associated with changes in foraging activity and dominance patterns in ants (Albrecht & Gotelli, 2001; Cerdá et al., 1998; Menke & Holway, 2006), the compelling evidence relating ant functional diversity to productivity (Arnan et al., 2014) makes it difficult to generalize about this issue. Thus, the role of niche specialization (e.g., different temperature preferences) or niche overlap (e.g., similar resource needs) in explaining the functional packing of *Pheidole* assemblages remains unresolved.

A third explanation for the ecological packing is that the morphological traits used here are weakly related to *Pheidole* ecological niches and, therefore, functional packing is unlikely. This alternative is however questionable given the strong evidence linking ant morphological traits with physiological (Arnan & Blüthgen, 2015; Arnan et al., 2014; Bishop et al., 2016), behavioral (Hurlbert, Ballantyne, & Powell, 2008; Medan & Josens, 2005), and life‐history traits (Weiser & Kaspari, 2006). Most likely, the similarities in morphological traits among *Pheidole* species do reflect similar ecological adaptations, which ultimately indicate that increased niche packing rather than niche displacement underlies the diversity pattern of *Pheidole* assemblages in Neotropical savannas. Overall, our findings lend support to the growing evidence that richness patterns in the tropics result from co‐occurrence of species with similar traits (Pigot, Trisos, & Tobias, 2016; Safi et al., 2011), reinforcing the view that communities are not saturated with species (Mateo, Mokany, & Guisan, 2017).

Although not directly addressed here, evolutionary processes can also have played a role in shaping functional richness patterns of *Pheidole* assemblages. A recent work shows that *Pheidole* evolved similar patterns of both climate‐richness association and body‐size distribution across different regions worldwide (Economo et al., 2015). Shared ancestry and historical contingency played a minor role in shaping *Pheidole* diversity, whereas a greater net diversification rate in warm–wet regions might explain *Pheidole* richness patterns (Economo et al., 2015). The greater functional packing of *Pheidole* assemblages in the southeastern Cerrado could reflect the re‐evolution of similar phenotypes there. Indeed, there is evidence for heterogeneity in diversification rates among ants (Pie & Tschá, 2009), including differences in the evolution of size and shape in *Pheidole* (Pie & Tschá, 2013). However, the factors that changed the relationship between diversification rates and ecological traits remain unclear.

In conclusion, we have addressed patterns of functional richness of a dominant ant genus in one of the most threatened tropical savannas of the planet. Our analyses reveal a tendency to find functionally packed *Pheidole* assemblages, particularly in species‐rich regions and at coarser spatial scales. According to our findings, the scale dependency of functional richness is potentially linked to the importance of morphometric traits at finer spatial scales, which may lose relevance at coarser spatial scales. Although competition has been traditionally considered the reigning paradigm in ant ecology (Cerdá et al., 2013), our findings indicate that environmental filtering plays a comparatively greater role in shaping ant assemblages in Neotropical savannas.

## CONFLICT OF INTEREST

The authors declare that they have no conflict of interest.

## AUTHOR CONTRIBUTIONS

MRM, KN, and HLV conceived the ideas and designed the methodology; KN, HLV, JM, and RP performed the field collections and sorted the ant specimens; MRM, KN, and HLV analyzed the data; and MRM led the writing. All authors contributed in the form of discussions and suggestions and approved the final manuscript.

## Data Availability

Functional data for *Pheidole* assemblages and the R‐scripts generated for this study are available at in the Dryad Digital Repository https://doi.org/10.5061/dryad.31201jg (Moura et al., 2019).
