# Supplementary material for: Functional richness shows spatial scale dependency in Pheidole ant assemblages from Neotropical savannas
Source: Ecol Evol. 2019 Sep 27;9(20):11734–41. doi: 10.1002/ece3.5672 (PMC6822040; doi:10.1002/ece3.5672)
Supplement: Supplementary file 1 [file ECE3-9-11734-s001.docx]

**Supporting Information**

Neves, K., Moura, M.R., Maravalhas, J., Pacheco, R., Pie, M.R., Schultz, T.R. & Vasconcelos, H.L. (2019) Functional richness shows spatial scale dependency in *Pheidole* ant assemblages from Neotropical savannas. Ecology & Evolution.

Appendix S1 – Additional table.

Table S1. List of the *Pheidole* species/morphospecies collected in this study. Columns below inform the number of times each taxon was recorded and the number of sites in which it was found. Morphospecies codes are those from the ant collection of the Federal University of Uberlândia in Brazil.

| **Species/Morphospecies** | **Number of records** | **Number of sites with occurrences** |
| --- | --- | --- |
| 1. *Pheidole aberrans* | 1 | 1 |
| 1. *Pheidole ambigua* | 31 | 9 |
| 1. *Pheidole aper* | 4 | 1 |
| 1. *Pheidole cavifrons* | 3 | 1 |
| 1. *Pheidole cyrtostela* | 26 | 6 |
| 1. *Pheidole fracticeps* | 419 | 29 |
| 1. *Pheidole gertrudae* | 70 | 11 |
| 1. *Pheidole jelskii* | 60 | 11 |
| 1. *Pheidole obscurithorax* | 3 | 2 |
| 1. *Pheidole oxyops* | 215 | 17 |
| 1. *Pheidole radoszkowskii* | 179 | 4 |
| 1. *Pheidole rufipilis* | 34 | 9 |
| 1. *Pheidole schwarzmaieri* | 16 | 4 |
| 1. *Pheidole scolioceps* | 5 | 4 |
| 1. *Pheidole* sp. nr. *tetrica* | 73 | 6 |
| 1. *Pheidole subarmata* | 14 | 6 |
| 1. *Pheidole susannae* | 18 | 3 |
| 1. *Pheidole synarmata* | 3 | 3 |
| 1. *Pheidole triconstricta* | 499 | 29 |
| 1. *Pheidole vafra* | 5 | 2 |
| 1. *Pheidole* sp. 01 | 7 | 5 |
| 1. *Pheidole* sp. 03 | 66 | 16 |
| 1. *Pheidole* sp. 06 | 1 | 1 |
| 1. *Pheidole* sp. 07 | 36 | 13 |
| 1. *Pheidole* sp. 08 | 20 | 9 |
| 1. *Pheidole* sp. 09 | 37 | 6 |
| 1. *Pheidole* sp. 10 | 36 | 14 |
| 1. *Pheidole* sp. 12 | 18 | 10 |
| 1. *Pheidole* sp. 13 | 46 | 7 |
| 1. *Pheidole* sp. 14 | 14 | 6 |
| 1. *heidole* sp. 15 | 191 | 22 |
| 1. *Pheidole* sp. 18 | 3 | 2 |
| 1. *Pheidole* sp. 19 | 2 | 2 |
| 1. *Pheidole* sp. 23 | 63 | 15 |
| 1. *Pheidole* sp. 25 | 5 | 3 |
| 1. *Pheidole* sp. 26 | 15 | 4 |
| 1. *Pheidole* sp. 27 | 15 | 3 |
| 1. *Pheidole* sp. 28 | 1 | 1 |
| 1. *Pheidole* sp. 29 | 1 | 1 |
| 1. *Pheidole* sp. 30 | 1 | 1 |
| 1. *Pheidole* sp. 33 | 54 | 11 |
| 1. *Pheidole* sp. 37 | 15 | 8 |
| 1. *Pheidole* sp. 38 | 25 | 9 |
| 1. *Pheidole* sp. 40 | 31 | 10 |
| 1. *Pheidole* sp. 41 | 6 | 2 |
| 1. *Pheidole* sp. 43 | 4 | 22 |
| 1. *Pheidole* sp. 44 | 15 | 2 |
| 1. *Pheidole* sp. 45 | 1 | 1 |
| 1. *Pheidole* sp. 47 | 2 | 2 |
| 1. *Pheidole* sp. 50 | 9 | 6 |
| 1. *Pheidole* sp. 51 | 7 | 3 |
| 1. *Pheidole* sp. 52 | 28 | 10 |
| 1. *Pheidole* sp. 53 | 13 | 5 |
| 1. *Pheidole* sp. 54 | 1 | 1 |
| 1. *Pheidole* sp. 55 | 3 | 1 |
| 1. *Pheidole* sp. 56 | 3 | 2 |
| 1. *Pheidole* sp. 58 | 1 | 1 |
| 1. *Pheidole* sp. 64 | 1 | 1 |
| 1. *Pheidole* sp. 65 | 18 | 4 |
| 1. *Pheidole* sp. 66 | 6 | 1 |
| 1. *Pheidole* sp. 69 | 6 | 6 |
| 1. *Pheidole* sp. 72 | 8 | 7 |
| 1. *Pheidole* sp. 77 | 4 | 2 |
| 1. *Pheidole* sp. 78 | 26 | 7 |
| 1. *Pheidole* sp. 79 | 1 | 1 |
| 1. *Pheidole* sp. 80 | 22 | 4 |
| 1. *Pheidole* sp. 82 | 6 | 2 |
| 1. *Pheidole* sp. 83 | 6 | 3 |
| 1. *Pheidole* sp. 84 | 2 | 2 |
| 1. *Pheidole* sp. 88 | 10 | 6 |
| 1. *Pheidole* sp. 90 | 1 | 1 |
| 1. *Pheidole* sp. 91 | 8 | 1 |
| 1. *Pheidole* sp. 92 | 6 | 1 |
| 1. *Pheidole* sp. 93 | 8 | 1 |
| 1. *Pheidole* sp. 94 | 3 | 2 |
| 1. *Pheidole* sp. 95 | 1 | 1 |
| 1. *Pheidole* sp. 97 | 4 | 1 |
| 1. *Pheidole* sp. 98 | 3 | 2 |
| 1. *Pheidole* sp. 101 | 1 | 1 |
| 1. *Pheidole* sp. 102 | 1 | 1 |
| 1. *Pheidole* sp. 103 | 1 | 1 |
| 1. *Pheidole* sp. 104 | 1 | 1 |
| 1. *Pheidole* sp. 105 | 2 | 2 |
| 1. *Pheidole* sp. 106 | 1 | 1 |
| 1. *Pheidole* sp. 107 | 1 | 1 |
| 1. *Pheidole* sp. 108 | 4 | 1 |
| 1. *Pheidole* sp. 109 | 1 | 1 |
| 1. *Pheidole* sp. 110 | 3 | 1 |
| 1. *Pheidole* sp. 122 | 1 | 1 |
| 1. *Pheidole* sp. 123 | 1 | 1 |
| 1. *Pheidole* sp. 124 | 1 | 1 |
| 1. *Pheidole* sp. 125 | 1 | 1 |
| 1. *Pheidole* sp. 126 | 1 | 1 |
| 1. *Pheidole* sp. 127 | 1 | 1 |
| 1. *Pheidole* sp. 128 | 1 | 1 |
| 1. *Pheidole* sp. 129 | 1 | 1 |
| 1. *Pheidole* sp. 130 | 1 | 1 |
| 1. *Pheidole* sp. 131 | 1 | 1 |
